# Supplementary material for: Low serum 25-hydroxyvitamin D status in the pathogenesis of stress fractures in military personnel: An evidenced link to support injury risk management
Source: PLoS One. 2020 Mar 24;15(3):e0229638. doi: 10.1371/journal.pone.0229638 (PMC7092979; doi:10.1371/journal.pone.0229638)
Supplement: S1 Table — aMedian (IQR) bMean (SE). (DOCX) [file pone.0229638.s001.docx]

**S1 Table. Baseline characteristics of stress fracture cases available and unavailable for follow-up.**

|  |  | Available  (*n* = 51) | Unavailable  (*n* = 65) | p |
| --- | --- | --- | --- | --- |
| Age (years)^a^ |  | 20 (6) | 19 (3) | 0.563 |
| Height (m)^b^ |  | 1.77 (0.008) | 1.75 (0.008) | 0.156 |
| Weight (kg)^b^ |  | 72.57 (1.08) | 72.05 (0.945) | 0.977 |
| VO2 max (ml kg^-1^ min^-1^)^a^ |  | 52.2 (4.0) | 51.1 (3.8) | 0.003 |
| Current smoker (%) |  | 10 (19.6%) | 7 (10.8%) | 0.182 |

^a^Median (IQR) ^b^Mean (SE)
