# Supplementary material for: Low serum 25-hydroxyvitamin D status in the pathogenesis of stress fractures in military personnel: An evidenced link to support injury risk management
Source: PLoS One. 2020 Mar 24;15(3):e0229638. doi: 10.1371/journal.pone.0229638 (PMC7092979; doi:10.1371/journal.pone.0229638)
Supplement: S2 Table — aMedian (IQR) bMean (SE). (DOCX) [file pone.0229638.s002.docx]

**S2 Table. Baseline characteristics of controls available and unavailable for follow-up.**

|  |  | Available (*n* = 141) | Unavailable (*n* = 1,378) | p |
| --- | --- | --- | --- | --- |
| Age (years)^a^ |  | 20 (4) | 20 (4) | 0.026 |
| Height (m)^b^ |  | 1.78 (0.005) | 1.77 (0.002) | 0.189 |
| Weight (kg)^b^ |  | 75.45 (0.630) | 74.66 (0.227) | 0.308 |
| VO_2max_ (ml kg^-1^ min^-1^)^a^ |  | 52.65 (4.3) | 51.9 (4) | 0.061 |
| Current smoker (%) |  | 14 of 140 (10%) | 152 of 1299 (11.7%) | 0.549 |

^a^Median (IQR) ^b^Mean (SE)
