# Supplementary material for: Low serum 25-hydroxyvitamin D status in the pathogenesis of stress fractures in military personnel: An evidenced link to support injury risk management
Source: PLoS One. 2020 Mar 24;15(3):e0229638. doi: 10.1371/journal.pone.0229638 (PMC7092979; doi:10.1371/journal.pone.0229638)
Supplement: S3 Table — aMedian (IQR) bMean (SE). (DOCX) [file pone.0229638.s003.docx]

**S3 Table. Baseline participant characteristics across vitamin D receptor (VDR) genotypes.**

|  |  | VDR *FF* (*n* = 67) | VDR *f** (*n* = 125) | p |
| --- | --- | --- | --- | --- |
| Age (years)^a^ |  | 20 (4) | 19.5 (6) | 0.368 |
| White ethnicity (%) |  | 66 (98.5%) | 123 (98.4%) | 0.270 |
| Height (m)^b^ |  | 1.77 (.0067) | 1.78 (.0053) | 0.709 |
| Weight (kg)^b^ |  | 74.15 (0.88) | 74.80 (0.66) | 0.558 |
| VO2_max_ (ml kg^-1^ min^-1^)^a^ |  | 52.2 (3.9) | 52.8 (4.6) | 0.792 |
| Alcohol intake (%) n = 63 vs 112 | 0 | 8 (12.7%) | 19 (17.0%) | 0.736 |
|  | 1 – 10 units | 39 (61.9%) | 61 (54.5%) |  |
|  | 11 – 20 units | 13 (20.6%) | 23 (20.5%) |  |
|  | >20 units | 3 (4.8%) | 9 (8.0%) |  |
| Current smoker (%) |  | 12 (17.9%) | 32 (25.6%) | 0.227 |
| Weight bearing activity index (%) 62 vs 122 | 1 (0-19) | 18 (29.0%) | 38 (31.1%) | 0.717 |
|  | 2 (20-99) | 21 (33.9%) | 46 (37.7%) |  |
|  | 3 (>100) | 23 (37.1%) | 38 (31.1%) |  |

^a^Median (IQR) ^b^Mean (SE)
