# Supplementary material for: Low serum 25-hydroxyvitamin D status in the pathogenesis of stress fractures in military personnel: An evidenced link to support injury risk management
Source: PLoS One. 2020 Mar 24;15(3):e0229638. doi: 10.1371/journal.pone.0229638 (PMC7092979; doi:10.1371/journal.pone.0229638)
Supplement: S4 Table — (DOCX) [file pone.0229638.s004.docx]

**S4 Table. Start of training across vitamin D receptor (VDR) genotypes.**

|  | VDR *FF* (*n* = 50) | VDR *f** (*n* = 99) | p |
| --- | --- | --- | --- |
| Spring (%) | 16 (32.0) | 30 (30.3) | 0.36 |
| Summer (%) | 5 (10.0) | 9 (9.1) |  |
| Autumn (%) | 11 (22.0) | 35 (35.4) |  |
| Winter (%) | 18 (36.0) | 25 (25.3) |  |
