# Supplementary material for: Low serum 25-hydroxyvitamin D status in the pathogenesis of stress fractures in military personnel: An evidenced link to support injury risk management
Source: PLoS One. 2020 Mar 24;15(3):e0229638. doi: 10.1371/journal.pone.0229638 (PMC7092979; doi:10.1371/journal.pone.0229638)
Supplement: S5 Table — (DOCX) [file pone.0229638.s005.docx]

**S5 Table. Logistic regression for stress fracture risk in propensity matched cases and controls – vitamin D receptor (VDR) genotype x baseline vitamin D interaction.**

| VDR genotype | OR per 1 SD increase in baseline vitamin D | P |
| --- | --- | --- |
| *FF*  *Ff*  *ff* | 1.76 (0.75-4.14)  0.70 (0.38-1.29)  0.29 (0.05-1.86) | 0.04 (trend) |
| *FF*  *f** | 1.71 (0.73-4.02)  0.62 (0.36-1.09) | 0.05 |
