# Supplementary material for: Low serum 25-hydroxyvitamin D status in the pathogenesis of stress fractures in military personnel: An evidenced link to support injury risk management
Source: PLoS One. 2020 Mar 24;15(3):e0229638. doi: 10.1371/journal.pone.0229638 (PMC7092979; doi:10.1371/journal.pone.0229638)
Supplement: S6 Table — (DOCX) [file pone.0229638.s006.docx]

**S6 Table. Logistic regression for stress fracture risk after multiple imputations – vitamin D receptor (VDR) genotype x baseline vitamin D interaction.**

| VDR genotype | OR per 1 SD increase in baseline vitamin D | P |
| --- | --- | --- |
| *FF*  *Ff*  *ff* | 1.57 (0.71-3.47)  0.84 (0.47-1.51)  0.37 (0.11-1.26) | 0.05 (trend) |
| *FF*  *f** | 1.55 (0.72-3.37)  0.69 (0.42-1.14) | 0.08 |
